# Effectiveness of Oral Nutritional Supplementation for Older Women after a Fracture: Rationale, Design and Study of the Feasibility of a Randomized Controlled Study

**DOI:** 10.1186/1471-2318-11-32

**Published:** 2011-06-10

**Authors:** Ian D Cameron, Susan E Kurrle, Cesar Uy, Keri A Lockwood, Lydia Au, Frederieke G Schaafsma

**Affiliations:** 1 Rehabilitation Studies Unit, Sydney Medical School, University of Sydney, Australia; 2 Division of Rehabilitation and Aged Care, Hornsby Ku-ring-gai Health Service, Sydney, Australia; 3 Department of Geriatrics, Alexandra Hospital, Singapore

## Abstract

**Background:**

Malnutrition is a problem for many older people recovering from a hip and other major fractures. Oral supplementation with high calorie high protein nutrients is a simple intervention that may help older people with fractures to improve their recovery in terms of rehabilitation time, length of hospital stay and mortality. This paper reports a pilot study to test the feasibility of a trial initiated in a hospital setting with an oral supplement to older people with recent fractures.

**Method:**

A randomized controlled trial with 44 undernourished participants admitted to a hospital following a fracture. The intervention group (n = 23) received a high calorie high protein supplement for forty days in addition to their diet of choice. The control group (n = 21) received high protein milk during their hospital stay in addition to their diet of choice and their usual diet when discharged from hospital.

**Results:**

All participants were women and their mean age was 85.3 (± 6.1) years. Twenty nine (65%) participants had a hip fracture. At baseline no differences were measured between the two groups regarding their nutritional status, their cognitive ability or their abilities in activities of daily living. There were no significant differences between the intervention and control group with reference to nutritional or functional parameters at 40 day and 4 month follow-ups. Median length of stay in hospital was 18.0 days, with 12 participants being readmitted for a median of 7.0 days.

**Conclusion:**

It is feasible to perform a randomised trial in a hospital and community setting to test the effect of an oral high energy high protein supplement for older people. Due to the limited number of participants and incomplete adherence with use of the supplements no conclusion can be drawn about the efficacy or effectiveness of this intervention.

## Background

The risk of malnutrition is increased in older people and it has been shown to have important effects on recovery in a broad range of conditions [1]. Malnutrition has been associated with impaired immune response, impaired muscle and respiratory function, delayed wound healing, overall increased complications, longer rehabilitation, greater length of hospital stay and increased mortality [2]. Older people with fractures are a particularly vulnerable group. Not only is there a high chance of these patients being malnourished upon admission [3], but a further decline in their nutritional state due to surgery and hospitalization is a serious risk [4]. Various initiatives, of which the majority includes supplementation, have been undertaken to help improve the nutritional state for those older people being admitted to the hospital [1,5]. However, the effects so far are questionable. The current Cochrane Review on nutritional supplementation for hip fracture aftercare in older people concluded that the there is only weak evidence for the effectiveness of protein and energy feeds, and more high quality randomized trials are needed [6]. No clear evidence was given that malnourished older patients benefit more than those who are not malnourished. It has been suggested that low compliance to oral supplementation may be a problem due to low palatability [6]. On the other hand, advantages of oral supplements are that the intervention itself is rather simple, takes only a short period of time, and is likely to be perceived as a relevant intervention by older people recovering from a fracture. These advantages should help improve compliance to the intervention [7-10].

The objectives of this pilot study are, firstly, to study the feasibility of a randomized trial initiated in the hospital setting. Secondly, to test the effectiveness of oral supplementation to malnourished older people with a fracture. We investigate the effectiveness of a high calorie, high protein nutritional supplement in terms of changes in recovery rate as measured by abilities in activities of daily living (ADL), and nutritional status.

## Method

### Participants

Patients admitted to Hornsby Ku-ring-gai hospital (a general hospital in Northern Sydney, Australia) from February 2000 to January 2003 with hip or other fractures were asked to participate within 5 days of admission. Due to limited funding and staff changes not all patients were approached, but approximately 10% to 20% of the patients screened satisfied the inclusion criteria. Inclusion criteria included: female aged over 70 with moderate or severe protein energy malnutrition (PEM). PEM was defined as moderate if the mid-upper arm circumference (MUAC) was less than the 10th percentile for age and gender, or if the pre-surgery serum albumin concentration was lower than or equal to 35 g/L [11]. Malnutrition was considered severe if both these criteria were met. Patients were excluded if they had diagnosed metastatic cancer, chronic renal failure or hepatic failure. After screening for their nutritional status, and obtaining informed consent from patients themselves or their person responsible, patients were randomized to either a supplement or usual care group. This study was approved by the Research Ethics committee of the Hornsby Ku-ring-gai Hospital.

The randomization sequence was derived from a random numbers table and the allocation was recorded on a card sealed in a sequentially numbered opaque envelope. Randomization was stratified for hip or other fracture, and extent of undernutrition. Once informed consent was given, the research nurse opened the next numbered envelope and informed the participant of the group to which they had been randomized.

### Intervention

The participants in the intervention group were asked to drink one pack of supplement per day from when oral intake was resumed after surgery, or from enrolment in subjects who had no surgical treatment. The supplementation was to be continued for forty days on a once daily basis. During the trial there was a change in supplement because the Sustagen Hospital Plus originally used in the study was no longer available in Australia. This change occurred after the first 12 months of recruitment when 11 participants had been enrolled. After taste testing and nutrient composition comparisons, Novasource was agreed to be acceptable and replaced Sustagen Hospital Plus (nutrients details of the supplements can be found in Additional File 1). If during the trial period of 40 days participants were discharged from the hospital they were given the remaining nutritional supplements with instructions to keep drinking them as discussed. Participants in the control group were given a high protein diet (with high protein milk) during their hospital stay because this was standard treatment at the study hospital and it was judged not to be ethical to withdraw this. After discharge they could follow their normal diet. Both groups received the same rehabilitation programs.

### Outcome measurements

Measurements took place at 1) baseline; which was after admission to hospital and before surgery, 2) after 40 days and 3) after 4 months. Baseline measures included weight, height, mid upper-arm circumference (MUAC), grip strength, serum albumin (g/L), activities of daily living as assessed by the Barthel Index [12], Short Portable Mental Status Questionnaire (SPMSQ) [13] to assess participant's cognitive status, and the Charlson Index to evaluate the presence of co morbidities [14]. The participant's knee height was used to calculate the estimated actual height so that body mass index (BMI) could be calculated [15].

Weight and height at baseline were obtained via self-report, or from the family carer, if possible. Although, it was realized that self-report of weight may not be the most accurate method this way was considered most practical [16,17]. Mid upper-arm circumference (MUAC) to the nearest millimeter was measured in the non dominant arm using a fiberglass tape at baseline. MUAC norms reported by Falciglia et al. were used as a reference when assessing MUAC measurement [18]. Grip strength in the dominant arm was measured with a hand-grip dynamometer (Jamar), using three trials with the highest reading recorded [19]. The Barthel Index was used to assess disability status and includes 15 self-care, sphincter-control, and mobility factors. A Barthel score of 40 or less is defined as very severely dependent; score of 60 or less defined as markedly dependent while score of 61-80 demonstrates less need for assistance [12].

The measurements were repeated at 40 days and 4 months follow up by a research nurse who was masked to the participants' treatment allocation. In addition after 40 days and after 4 months the gait speed of participants was measured. The time required for subjects to walk 2.44 m was used to calculate gait speed [20].

Details of complications and length of hospital stay were obtained from the subjects' medical records. Ascertainment of these complications was completed by the research nurse. Readmission to hospital and mortality were also recorded.

### Statistical analysis

The data analysis was performed using SPSS 16.0 (SPSS Inc., Chicago, IL, USA). P < 0.05 was considered statistically significant. For descriptive purposes, mean values and standard deviations were used for normal distributed data, and range and medians for data with a skewed distribution. For comparison between groups, student's *t*-test was used or a Mann-Whitney *U*-test. For comparison between follow-up measurements, a paired *t*-test or Wilcoxon signed rank test was used for each group. Subgroup analyses were performed for participants with hip fractures and for participants with lower cognitive abilities based on the SPMSQ test. A backward stepwise multiple linear regression analysis was performed to study the effects of multiple independent variables on the studied continuous variables.

## Results

We included 44 participants with 27 participants (65%) having a hip fracture - equally divided over the two groups. Other fractures included pelvis, humerus, or femoral shaft fractures. Baseline characteristics showed no differences between the two groups (see Table 1). Thirty nine percent of participants (7 in control group, and 10 in intervention group) were severely undernourished with both serum albumin < 35 g/l and MUAC less than the 10^th ^percentile for women over 70 years. Fifty percent of participants (10 in control group, and 12 in intervention group) were undernourished with serum albumin < 35 g/l, and 11% of participants (4 in control group, and 1 in intervention group) were considered undernourished with MUAC less than the 10^th ^percentile for women over 70 years.

**Table 1:** Baseline characteristics of study participants

	Control group (n = 21)	Intervention group (n = 23)	T-test p- value
Age (years) (SD)	87.1 (6.2)	83.7 (5.6)	0.07

SPMSQ (mean n of errors (SD))*	5.1 (3.8)	5.8 (3.7)	0.52

Charlson Index (mean score (SD))	1.7 (1.8)	1.5 (1.1)	0.67

Weight (kg) (SD)**	50.2 (11.8)	50.4 (6.0)	0.80

BMI (SD) **	21.5 (4.0)	21.5 (2.8)	0.95

MUAC (cm) (SD)***	23.6 (2.6)	24.2 (3.1)	0.53

Grip (kg) (SD)	11.0 (5.8)	13.7 (6.8)	0.18

Albumin (g/l) (SD)	31.8 (5.4)	31.1 (5.0)	0.69

Barthel Index (SD)§	76.6 (21.6)	74.1 (23.4)	0.73

* SPMSQ= Short Portable Mental Status Questionnaire

** based on only 12 participants in control group, 11 participants in intervention group

*** MUAC= mid upper arm circumference

§ Estimated Barthel Index prior to fracture

During the supplementation of 40 days there were two participants in the intervention group who withdrew and after 4 months another participant in this group withdrew. One participant died during the 4 months. Compliance of participants in the intervention group was unclear as no objective measure of this was available. The research staff mentioned that main reasons participants reported for being non compliant at home were that they just did not have the capacity to drink the supplement because they found it too filling, or it ended up making them feel nauseated, or they did not like the taste because it was too sweet.

Diet history of preadmission intakes was obtained from nine subjects, and the diet history of 40 days after fractures were obtained from seven subjects. No significant differences were found between the control group and intervention group at base line and 40 days after fractures in terms of energy and protein intake. Both the control and intervention subjects decreased their energy intake and protein intake after their fractures.

Table 2 shows the results of the first follow up after 40 days of supplementation of a high calorie, high protein nutritional supplement to the intervention group. After 40 days no differences could be measured between the two groups in any of the outcomes. Table 2 also shows the results of the second follow up after 4 months. Again, no differences could be measured between the two groups in any of the outcomes.

**Table 2:** Results after 40 days and after 4 months

	Control group after 40 days (n = 21)	Intervention group after 40 days (n = 23)	Statistical test† p-value	Control group after 4 months (n = 21)	Intervention group after 4 months (n = 23)	Statistical test† p-value
Weight (kg) (SD)	48.6 (8.5)	47.7 (7.8)	0.74	46.3 (7.6)	46.9 (7.5)	0.81

BMI (SD)	21.5 (4.0)	20.5 (3.1)	0.39	20.5 (3.8)	20.2 (2.9)	0.81

MUAC (cm) (SD)*	23.7 (3.0)	24.3 (2.8)	0.52	23.3 (3.3)	24.1 (2.7)	0.41

Grip strength (kg) (SD)	12.3 (6.2)	14.7 (5.6)	0.19	12.4 (5.6)	14.8 (5.6)	0.20

Albumin (g/l) (SD)	33.2 (4.4)	33.9 (3.9)	0.62			

Gait velocity (m/sec) (SD)**	0.19 (0.21)	0.20 (0.21)	0.89	0.21 (0.24)	0.27 (0.24)	0.48

Barthel Index (SD)	56.0 (35.0)	66.3 (29.5)	0.31	58.0 (35.8)	60.3 (35.8)	0.84

* MUAC= mid upper arm circumference

** Gait velocity measured as how many seconds to finish 2.44 m walk, with those unable to walk calculated as 0 m/sec.

† T-test or Mann-Whitney Test between control and intervention group

On average, weight declined steadily for both groups during the intervention period: from 51.1 kg at baseline to 48.7 kg after 40 days (for n = 22, p = 0.04). After 4 months weight declined slightly further from 47.9 kg to 47.0 kg after 4 months (n = 33, p = 0.18). We found only 2 participants in the control group and 1 in the intervention group with increased weight after 4 months. The mid upper arm circumference of participants showed no significant change over time for both groups. Serum albumin increased in both groups from an average of 31.5 g/l at baseline to 33.5 g/l after 40 days (n = 41, p = 0.035). Grip strength had also increased after 4 months for both groups, but not significantly. Half of the participants in both groups were unable to walk without aid after 40 days and were still unable after 4 months. Gait velocity increased non- significantly between the 40 days and 4 months measurement for all participants (n = 39, p = 0.098). Mean scores on the Barthel Index dropped significantly from the estimated pre-injury score to that after 40 days (n = 41, p < 0.000). No significant change in the Barthel Index was measured between 40 days and 4 months follow up. We found 3 participants in the control group with increased Barthel Index (mean = 6.7 points), and 7 participants in the intervention group (mean 12.3 points) after 4 months.

No differences were measured in initial length of stay and after re-admission between groups. Median length of stay in hospital for the control group was 14.0 days (range 5-56). Six participants were readmitted during the study period for a median of 9.5 days (range 1-128). Median length of stay for the intervention group in hospital was 23.5 days (range 1-67) and six participants were readmitted with a median of 7.0 days (range 1-28). Thirteen participants suffered at least one complication during the trial (8 in the control group and 5 in the intervention group) such as urinary tract infections (6 times), pleural effusion (2 times), wound infection (1 time), pulmonary oedema (1 time), delirium (1 time) and a decubitus ulcer (1 time).

We performed a backward stepwise multiple linear regression with change in serum albumin, measured from baseline to 40 days, as dependent variable and age, hip fracture, baseline MUAC and total length of hospital stay (within study period) as independent variables. Total length of hospital stay was independently predictive of the change in serum albumin (R^2 ^= 0.142, F = 6.39, p = 0.016), meaning that the longer the stay in hospital the greater the increase in serum albumin. When change in gait velocity was analyzed as a dependent variable, using similar independent variables, only greater age was independently predictive of slower gait velocity (R^2 ^= 0.127, F = 5.37, p = 0.026).

Subgroup analysis for participants with a hip fracture again showed no differences between the two groups. However, grip strength was slightly better in the intervention group than in the control group after 40 days (15.2 (± 6.2) vs 10.4 (± 6.2), p = 0.056) and the trend remained after 4 months (15.3 (± 6.0) vs 11.1 (± 6.0), p = 0.092). For participants with lower cognitive capacity measured as more than 4 errors in the Short Portable Mental Status Questionnaire no differences in results were measured between the two groups.

## Discussion

The use of a daily high calorie high protein energy supplement as a simple intervention in a randomized trial design for older people recovering from a fracture is feasible. However from the results of this small pilot study no significant benefits were measured for the intervention group in terms of better recovery, less complications, or better nutritional status after 4 months. Due to the small sample size and the uncertainty about compliance for both groups no conclusion can be drawn about these results.

The inclusion period for this trial was almost three years to recruit 44 eligible and willing older female participants who fulfilled our inclusion criteria of having a fracture and being undernourished. This is a long recruitment period for such a small number of participants, but we believe that with more staff and funding it would have been possible to include many more eligible participants in a shorter time frame. This could also have helped to overcome the possible issue regarding uncertainty with compliance. For future studies it would be valuable to record the participants overall consumption of the commercial or food supplement, the energy or protein intake provided and the proportion of nutritional requirement being met. Not just prescribing or receiving supplements but knowing if people are indeed reaching their nutritional goals is the critical factor.

For the control group it was the policy in the hospital to provide the patients with high protein milk, containing half the energy and protein of the intervention group. Also, for this group of patients it is uncertain to what extent they complied with the policy of the hospital. As the weight in both groups of patients declined and continued to decline future studies not only need to know exactly what amount of supplement was actually used, but also what the diet was during both hospital stay and after discharge. A compliance officer that would record nutritional intake for both groups is recommended for future studies.

One of our inclusion criteria on nutritional status was based on the study by Falciglia et al. [18]. She calculated reference norms for the mid upper arm circumference based on older people living in the USA. It is possible that the Australian participants in this study appeared more undernourished compared to these US norms than if they had been compared to a different set of reference norms. There are various ways to define and to assess malnutrition as described by Milne et al. 2009 in their Cochrane Review [2]. We used the definition by Constans et al. and included 50% of participants based on reduced Albumin alone (with MUAC > 10th percentile for age and gender) [11]. Future studies using different definitions of malnutrition may find different effects of oral supplements. During the first 40 days when the majority of patients were still in hospital, serum albumin increased significantly in association with the high calorie, high protein nutritional supplement and/or the high protein milk provided. Nevertheless it appeared that weight decreased significantly during that same period. After 4 months, when the majority of participants were no longer in hospital, a further decline in weight was measured. This can in part be attributed to patients not being compliant with the supplements after discharge, or because the supplement provision was not adequate to address the actual requirements. Those patients with cognitive impairment who were discharged to nursing homes probably faced similar difficulties.

Both types of oral supplements used in this study were considered reasonably palatable by the research staff. However, during the intervention period a few complaints were received that participants actually did not like to drink the supplement because of its taste or because it was too filling. As it may be impossible to find an oral supplement that suits everybody, an alternative could be to use the strategy suggested by Miller et al. [21]. They provided oral supplements with 6 types of flavour and in different amounts depending on the energy requirement of the patient in a similar trial. On the other hand, this strategy resulted in a median compliance of 65% only, and it may be too impractical for hospitals to individualize the supplement to this extent.

The recent updated Cochrane Review on nutritional supplementation concluded that oral supplementation has no proven effect on post hip fracture mortality [6]. The authors of that review also concluded that oral supplementation may possibly reduce 'unfavourable outcomes' (death or complications) although there were no statistically significant effects reported. Consistent with these results, in this small pilot study no difference in number of complications could be measured between the two groups.

The majority of included studies in the Cochrane Review compared high protein supplement with a control situation, whereas in this study we compared a high protein and high energy supplement with a high protein supplement while participants were hospitalised. The difference in percentage protein between the supplement Sustagen Hospital Plus and the high protein milk is only 0.2 g per 100 ml and between the supplement Novasource 2.0 and the high protein milk 1.7 g per 100 ml. Therefore, in this trial the effect from the intervention would mostly be a result from the high energy of the supplement (a combination of the carbohydrates and fat) and the vitamins and minerals. The study by Botella-Carretero et al. who tested the effect of similar types of supplement as used in this study on normally nourished or mildly undernourished patients with a hip fracture also showed little difference in effect on the nutritional status [5]. However, that study did find that for those patients who underwent a surgical treatment and/or who had a longer hospital stay, both supplements could have a positive effect on serum albumin measured at discharge. In line with that study, we found that total length of hospital stay also had a positive effect on serum albumin after four months. Although it seems likely that during the hospital stay compliance with the use of prescribed oral supplements was higher than after discharge, this effect may also be a result of the standard procedure in hospital of providing high protein milk.

Strength of this pilot study is the randomized controlled design and the follow up time to measure the effect of oral supplementation for a longer time than just during hospital stay. The small number of participants due to the pilot character of this study may have resulted in a lack of effect in any of the outcome measures. Although research so far is unclear whether oral supplements reduce normal dietary intake, it could have been useful to analyse this information as well [2,21]. For future studies, we recommend that a similar study design is used with more participants and with more information about actual compliance and other dietary intake. In this study, standard procedure for older people admitted to this particular hospital was to provide high protein milk in addition to their normal hospital diet. Future trials need to take into account that general policies of hospitals may now include supplementation as standard for older people recovering from a fracture. This may complicate performing a randomized trial testing particularly the effect of oral supplementation in these settings. Based on trends in this pilot study it may require a smaller sample size to detect a difference in functioning than an impact on nutritional variables (see Table 2).

## Conclusion

Performing a randomized trial in a hospital setting to test the effect of an oral high energy high protein supplement for older people suffering from a fracture is feasible. No conclusion can be drawn about the effect of nutritional supplementation for this type of patients due to the limited number of participants and incomplete adherence with use of the supplements. Further randomized studies appropriately sized and with rigorous controls of the intervention are needed.

## Pre-publication history

The pre-publication history for this paper can be accessed here:

http://www.biomedcentral.com/1471-2318/11/32/prepub

## Supplementary Material

Additional File 1 **Appendix 1**. Nutrients Value for supplements. Click here for file
