# Supplementary material for: Effectiveness of Oral Nutritional Supplementation for Older Women after a Fracture: Rationale, Design and Study of the Feasibility of a Randomized Controlled Study
Source: BMC Geriatr. 2011 Jun 10;11:32. doi: 10.1186/1471-2318-11-32 (PMC3127759; doi:10.1186/1471-2318-11-32)
Supplement: Additional File 1 — Appendix 1. Nutrients Value for supplements. [file 1471-2318-11-32-S1.DOC]

# Appendix 1

Nutrients Value for supplements

1. Novasource 2.0

| Nutrients | Unit | Value per 237 mls | Per 100mls |
| --- | --- | --- | --- |
| Energy | Kcal | 475 kcal | 200.4 |
| Protein | G | 21.3 | 9.0 |
| Carbohydrates | G | 51 | 21.5 |
| Fat | G | 20.9 | 8.8 |
| Vitamins: |  |  |  |
| Vit A | μg | 375 | 158.2 |
| Vit D | μg | 2.5 | 1.05 |
| Minerals: |  |  |  |
| Ca | mg | 250 | 105.5 |
| Iron | mg | 4.5 | 1.90 |
| Phosphate | μg | 250 | 105.5 |

*N.B. The fat in Novasource comes from Canola oil and MCT*

*This is lactose and gluten free*

1. Sustagen Hospital Plus

| Nutrients | Unit | Value per 235ml | Per 100mls |
| --- | --- | --- | --- |
| Energy | Kcal | 352.5 | 150 |
| Protein | G | 17.6 | 7.5 |
| Carbohydrates | G | 44.2 | 18.8 |
| Fat | G | 11.7 | 5.0 |
| Vitamins: |  |  |  |
| Vit D | μg | 1.18 | 0.5 |
| Minerals: |  |  |  |
| Iron | mg | 3.8 | 1.6 |

N.B. This is lactose and gluten free

C. Hi protein Milk

| Nutrients | Unit | Value per 150ml | Per 100ml |
| --- | --- | --- | --- |
| Energy | Kcal | 194 | 129.3 |
| Protein | G | 11 | 7.3 |
| Carbohydrates | G | 18.75 | 12.5 |
| Fat | G | 8.3 | 5.5 |
